# Iron Metabolism and Immune Regulation

**DOI:** 10.3389/fimmu.2022.816282

**Published:** 2022-03-23

**Authors:** Shuo Ni, Yin Yuan, Yanbin Kuang, Xiaolin Li

**Affiliations:** ^1^ Department of Orthopedic Surgery and Shanghai Institute of Microsurgery on Extremities, Shanghai Jiaotong University Affiliated Sixth People’s Hospital, Shanghai, China; ^2^ State Key Laboratory for Diagnosis and Treatment of Infectious Diseases, the First Affiliated Hospital, School of Medicine, Zhejiang University, Hangzhou, China; ^3^ Department of Pulmonary Medicine, Shanghai Chest Hospital, Shanghai Jiao Tong University, Shanghai, China

**Keywords:** iron metabolism, immune regulation, macrophage polarization, neutrophils, NET, NK cell, B cell

## Abstract

Iron is a critical element for living cells in terrestrial life. Although iron metabolism is strictly controlled in the body, disturbance of iron homeostasis under certain type of condition leads to innate and adaptive immune response. In innate immunity, iron regulates macrophage polarizations, neutrophils recruitment, and NK cells activity. In adaptive immunity, iron had an effect on the activation and differentiation of Th1, Th2, and Th17 and CTL, and antibody response in B cells. In this review, we focused on iron and immune regulation and listed the specific role of iron in macrophage polarization, T-cell activation, and B-cells antibody response. In addition, correlations between iron and several diseases such as cancer and aging degenerative diseases and some therapeutic strategies targeting those diseases are also discussed.

## Highlights

1. Iron regulates macrophage polarizations.

2. Iron plays an important role in the functioning of neutrophils. Iron is involved in the formation of neutrophil extracellular traps (NETs). Transferrin secreted by human neutrophils promotes tumor metastasis.

3. Iron plays a pivotal role not only in the development and proliferation but also in the activation and function of NK cells when virus infection occurred.

4. Iron inhibits differentiation and activation of Th1, Th2, Th17, and Treg cells; on the other hand, iron as adjuvant promotes Th1 and Th2 cells immune response. Iron promotes CTL differentiation.

5. Targeting iron and immune regulation in cancer offered us new insight into promising therapies.

**Graphical Abstract:**
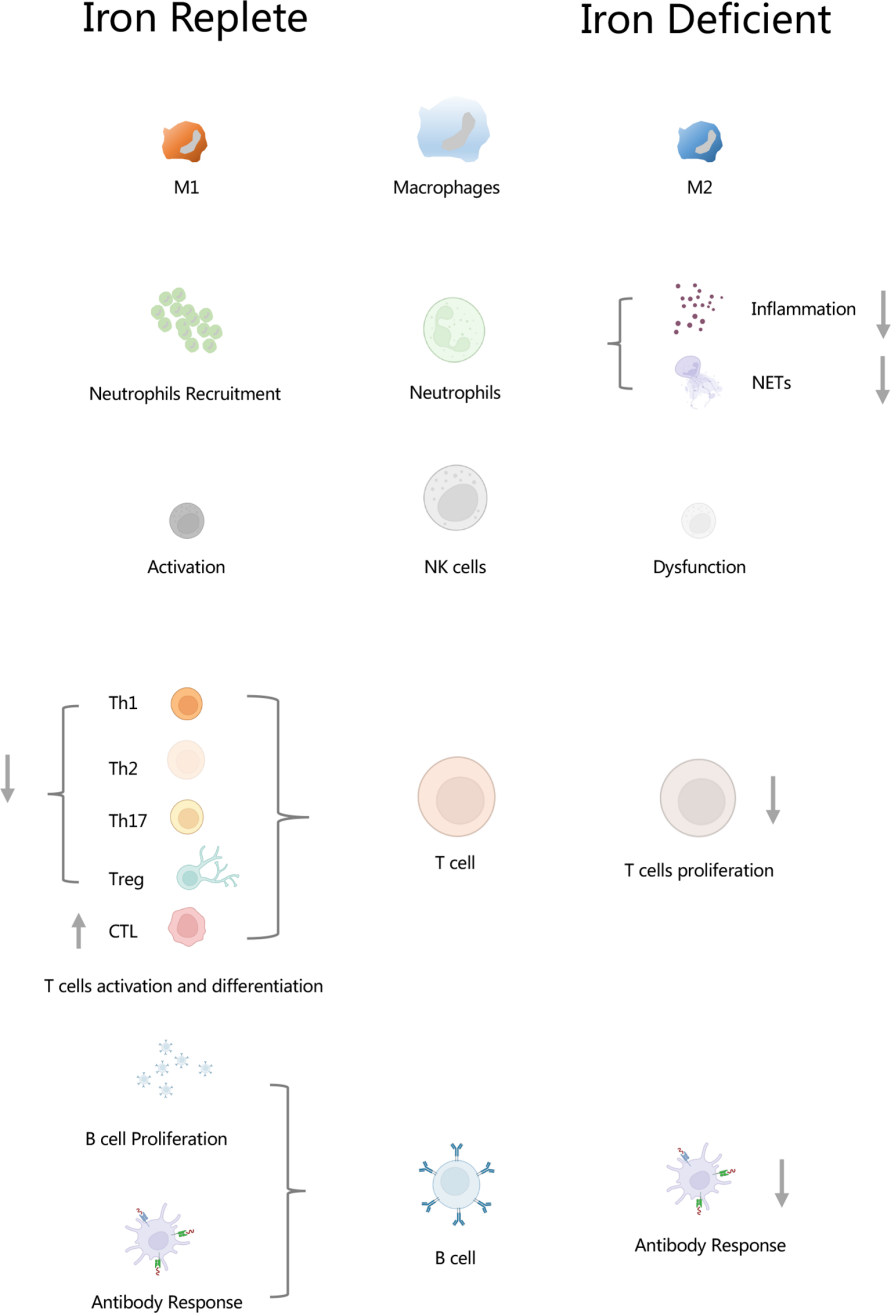
Iron regulates innate and adaptive immunity. In macrophages, iron regulates macrophage polarizations. In neutrophils, iron plays an important role in the functioning of neutrophils, and it is involved in the formation of neutrophil extracellular traps (NETs). In NK cells, iron is essential for the activation of NK cells. Iron inhibits differentiation and activation of Th1, Th2, Th17 and Treg cells, but it promotes CTL differentiation. Lack of iron resulted in inhibition of T cell proliferation. Iron increased B cell proliferation as well as antibody response. Insufficient of iron leaded to weaken antibody response. NETs, neutrophil extracellular traps; CTL, cytolytic T lymphocyte.

## Introduction

Iron, as a critical trace element, plays a critical role in terrestrial life. Multiple cellular activities such as synthesis of DNA, production of ATP, and respiration of mitochondrion are influenced by iron (1). As an essential factor in the production of enzymes and proteins, iron functions its role by exchanging electrons with multiple molecules (2). In this process, some highly reactive hydroxyl radicals (OH•) generated by Fenton reaction may result in the excess of reactive oxygen species (ROS) if they are not cleaned immediately (3). An excess of ROS leads to the injury of DNA, protein, lipid, and many other molecules (4). Therefore, iron plays a “double-edged sword” role in cells.

Clearly, iron is inherently an essential factor in the survival, development, and differentiation of cells, tissues, and organs. However, disruption of iron homeostasis is linked to the disorders of nervous, cardiovascular, and hematological systems (5). Indeed, multiple diseases including infection, cancer, and aging diseases appear to be associated with iron homeostasis (6–8). Notably, iron homeostasis also influences immune system, including innate and adaptive immunity (6). Recent studies reveal that targeting iron metabolism could be a potential method in immune-related disease (9, 10). Thus, investigation on the links between iron metabolism and immune regulation is therefore meaningful in clinical and basic research.

## Iron and Innate Immunity

Innate immunity acts as the first defense by natural mechanical barriers including skin, mucus, and gastric acid, protecting host from pathogens invasion. Several types of cells like macrophages, neutrophils, and dendritic cells participate in innate immunity. They respond rapidly once pathogens invade and activate inflammatory response immediately, followed by the initiation of adaptive immunity (11). Pathogen-specific motifs recognized by pattern-recognition receptors (PRRs), which are defined as pathogen-associated molecular patterns (PAMPs), induce innate immunity. In the same way, products from host damaged cells (e.g., extracellular ATP) recognized by PRRs are regarded as danger-associated molecular patterns (DAMPs) (11). Systemic and cellular iron level decreased significantly after pathogens invaded, which could be part of antibacterial mechanisms in the host (12). When infection occurs, the host will reduce the uptake of iron from the intestine and increase the expression of iron storage proteins. Then, the host will transfer iron from the plasma into ferritin, a protein that functions as a storing iron, and further reduce the concentration of free iron in host. This mechanism is known as “nutritional immunity,” which was induced by innate immunity under inflammation conditions (13, 14).

## Iron Regulates Macrophage Polarization

Macrophages show three different types of polarizations in response to microenvironment: un-activated M0 (un-activated), M1 (classically activated), and M2 (alternatively activated). M1 macrophages show a proinflammatory phenotype that secretes proinflammatory cytokines like TNF⁃α and IL⁃1β. M2 macrophages secrete TGF⁃β and PDGF. Moreover, M2 macrophages could be classified as several subtypes, namely, M2A, M2b, M2C, and M2d (15). M1 macrophages are prone to be with iron storage phenotype; this helps M1 macrophages to confer resistance to bacteria and tumor (2, 16). In contrast, M2 macrophages activated by IL-4 appear to be with the phenotype of releasing iron as a result of high expressions of CD163, CD94, iron export transporter, and low expression of ferritin (17). Release of iron promotes cell proliferation, matrix remodeling, and immune regulation, which is consistent with the function of M2 macrophages. Macrophages iron overload often leads to activation of M1 and induces expression of TNF α, IL-12p40, and CD163 (18). Notably, iron promotes M1 macrophage polarization under a certain type of conditions (5, 16, 19, 20); on the contrary, iron also promotes M2 polarization (21). These paradoxical findings reveal that macrophage showed heterogeneous phenotypes and dynamic populations (22, 23).

M1 polarization is characterized by the secretion of inflammatory cytokines (e.g., IL-6, TNF-a, etc.) and increase in expressions of some iron metabolism proteins like ferroprotin (FPN), transferrin receptor 1 (TfR1), CD163, ferritin, and HO-1, while M2 polarization appears to be with opposite changes in those proteins (24). Mechanistically, M1 polarization is usually followed by downregulation of FPN and upregulation of transferrin receptor 1, a membrane receptor that mediates holo-transferrin iron into cells (25). Similarly, some studies reveled that low expression of FPN and high expression of ferritin, a protein with a function of storing intracellular iron, could induce M1 polarizations (24). On the contrary, the upregulation of transferrin receptor and lipocalin usually leads to M2 polarization (24). Therefore, the polarization of macrophage is closely associated with iron context under different conditions, which means that when it comes to an iron replete condition, macrophage is usually prone to M1 polarization, while a deficiency in iron usually leads to M2 polarization. In addition, although an iron-deficient environment induced by iron chelator often leads to M2 polarization, few studies focused on the specific subtype of M2 under such conditions. It is therefore fascinating to explore the underlying mechanisms of iron on the state of polarization in macrophage (**Table 1**). Further investigations and explorations should be made on those aspects, and more attention is needed on the links between iron metabolism and macrophage polarization.

**Table 1:** Iron and macrophage polarization.

Iron level	Mφ	*in vitro*	Treatment	*in vivo*	Dosage and administration	Ref.
Replete	M1	RAW 264.7; human monocyte	FAC, 200 μM	C57BL/6 mice (male, 6–8 weeks old)	FAC, 1 g/kg (10 μl/g), i.p., 3 days	(26)
	M1	Mouse BMDM; Mouse microglial	Iron-dextran, 20 mM; RBCs (at a concentration of 10:1)	C57BL/6 mice (female, 6–8 weeks old)	Iron-dextran, 5 mg (5 mg of iron-dextran at 200 μl solution), i.p., 7 days	(19)
	M1	Human macrophage	Fe (III)-chloride, 10 μM	C57BL/6 mice (8–12 weeks old)	Iron-dextran, 5 mg (5 mg of iron-dextran at 200 μl solution), i.p., every 3 days for 21 days	(18)
	M1	RAW 264.7;	PEG-Fns(320 μM Fe)	Balb/c mice (female, 5 weeks old); Balb/c nude mice (female, 5 weeks old)	PEG-Fns 50μL (5 μmol Fe), intratumoral injection, every 7 days for 14 days; PEG-Fns 800 μmol Fe kg^-1^ at 200 μl solution, i.v, once	(20)
	M1	RAW 264.7; peritoneal macrophages;	Iron-induced macrophages after cryo-thermal therapy	Balb/c mice (female, 6-8 weeks old);	Cryo-Thermal Therapy Procedures	(27)
	M2	Human monocyte	FAC, 100 µM, 150 µM	Zebrafish	FAC, 100 µM, mfap4: tomato transgenic larvae, cells xenotransplantation injected into zebrafish, 24 hours	(21)
Deplete	M2	Human macrophage	DEF, 500 μM	Wistar Kyoto Rat (male, 16-weeks old)	DEF, 200 mg/kg/day in 0.1% carboxymethyl cellulose, oral gavage, 6 days	(28)
	M2	Human macrophage	N/A	C57BL/6 mice (8–12 weeks old)	DEF, 6 mg at 200 μl solution), i.p., every 3 days for 21 days	(18)
	M2	Mouse BMDM; RAW 264.7	DFO, 0-150 µM	N/A	N/A	(29)

FAC, ferric ammonium citrate; BMDM, bone marrow-derived macrophage; RBC, red blood cells; PEG-Fns, PEG-coated ferrihydrite nanoparticles; DEF, deferiprone. N/A means Not Applicable.

## Iron Regulates Neutrophils Recruitment and Inflammation

Neutrophils, as the most important cells in innate immunity, play a defensive role against microbial invasion. Iron-metabolism-related proteins (e.g., TfR1, FTH, FPN) are expressed in neutrophils, making it possible for neutrophils to uptake or release iron upon stimulations. Iron is an essential element in the functioning of neutrophils (30). Iron-dependent metalloprotein myeloperoxidase (MPO) in neutrophils had anti-microbial effect by its Fe^3+^/Fe^2+^ redox state (31). In addition, lipocalin-2 and lactoferrin, as iron-scavenging proteins secreted by neutrophils, chelate iron immediately as iron level increases (32, 33). Recent studies demonstrated that hepcidin, a peptide characterized by inducing degradation of FPN, which led to accumulation of intracellular iron, increases neutrophils recruitment by induction of CXCL1 (34). On the contrary, deferasirox, a Food and Drug Administration (FDA)-approved iron chelator, reduced neutrophil-mediated inflammation significantly (35), demonstrating that the depletion of iron *in vivo* reduces the amount and recruitment of neutrophils. However, direct evidence regarding iron regulating neutrophils recruitment and inflammation is scarce. Published studies showed that interactions between iron and neutrophils is a pathological consequence of different types of disease (34, 36–38). To the best our knowledge, the mechanism of iron regulating neutrophils recruitment and inflammation is most likely the chemokines secreted by certain types of cells influenced by iron, which means that the whole process is regulated by iron indirectly (34, 36–38). In necrotizing fasciitis, keratinocyte-secreted hepcidin promotes CXCL1 production, thus promoting neutrophils recruitment. Mechanistically, hepcidin induced FPN internalization and degradation, resulting in an increase in intracellular iron, which upregulates expression of CXCL1 in keratinocyte and subsequently promotes neutrophils recruitment (34). In some iron-overload disease, iron promotes neutrophils recruitment and inflammation by upregulating IL-1β (36). In Shiga-toxin-induced hemolytic–uremic syndrome, heme-scavenging proteins, haptoglobin, attenuated kidney platelet deposition and neutrophil recruitment, revealing the potential correlation between iron and neutrophil recruitment (37).

Recently, Vollger et al. indicated that the iron chelator deferoxamine (DFO) promotes the formation of neutrophil extracellular traps (NETs), which are closely related to antibacterial peptides, histones, and proteases in human neutrophils (39). This suggested that iron deficient environment enhances the antibacterial effect of neutrophils in infection. However, in sickle cell disease (SCD), DFO or the specific iron-binding protein apo-transferrin would prevent NET release (40). In addition, interestingly, some studies demonstrated that transferrin, a protein responsible for iron transporting, secreted by human and mouse neutrophils, promotes tumor metastasis (41).

Therefore, the specific role of iron in neutrophil is confusing; whether iron promotes neutrophil production or not, and the underlying mechanisms in iron-induced neutrophil recruitment and inflammation remain to be further explored in the future (**Table 2**).

**Table 2:** Iron and neutrophils.

Iron related factor	Experimental model	Treatment	Major findings	Ref.
Hepcidin	Necrotizing fasciitis	Hepcidin	Hepcidin is required for neutrophil response to bacterial infection	(34)
Iron chelator Deferasirox	Mice oropharyngeal candidiasis model	Deferasirox	Iron chelator Deferasirox reduces murine oropharyngeal candidiasis	(35)
Iron chelator deferoxamine (DFO)	Human neutrophils	Deferoxamine	DFO promotes the formation of neutrophil extracellular traps (NETs)	(39)
Iron chelator	Human neutrophils	DFO or iron-binding protein apotransferrin	Iron chelators prevent NET release	(40)
Iron chelator protein	Bacteria	metal-ion depletion	Calprotectin secreted by neutrophils had iron-sequestering function	(33)
Transferrin	4T1 tumor-bearing BALB/c mice	Anti-GM-CSF therapy	Transferrin secreted by human and mouse neutrophils promotes tumor metastasis	(41)

DFO, deferoxamine; NETs, neutrophil extracellular traps; GM-CSF, granulocyte-macrophage colony-stimulating factor.

## Iron and NK Cells

Activation of NK cells usually leads to the elimination of pathogens through releasing of several particles (e.g., granzyme and perforin), and the expression of TRAIL or FasL. These ligands serve as death receptor ligands and therefore induce target cell apoptosis. Cytokines such as interferon (IFN)-γ and tumor necrosis factor (TNF) produced by NK cells not only activate immune response but also make NK cells become sensitive to other cytokines (e.g., interleukin-2, IL-12, IL-15 and IL-18) (42, 43). Activated NK cells increase expression of transferrin receptor (CD71) (44, 45), whereas iron deficiency results in a lose function of NK cells (46). All of these indicate that iron is indispensable in the activation of NK cells.

Recently, Elisabeth et al. reported the close correlation between iron and NK cells activity. In their report, an increased absorption of iron was found followed by NK cells activation. Furthermore, they demonstrated that the subtypes of iron-absorption NK cells are CD27^+^ CD11b^+^ NK cells. Moreover, systematic low iron level influenced by hepcidin resulted in the suppression of NK cell activation and production of IFN-γ. Besides, they also showed that sufficient serum iron is critical to the metabolism of NK cells and their activity against virus infection (46). Accordingly, iron plays a pivotal role not only in the development and proliferation but also in the activation and function of NK cells when virus infection occurred.

## Iron and Adaptive Immunity

When it comes to clonal expansion of lymphocyte subsets, iron serves as a promoter in adaptive immune system (47, 48). Recent studies showed that adaptive T-cell immunity require serum iron by TfR1 (CD71) (49), highlighting the key role of CD71 in the uptake of iron. Immune deficiency characterized by impaired lymphocyte development and function is due to gene mutation of transferrin receptor 1 (CD71), which affects the internalization of iron-transferrin complex (50).

## Iron and T Cells

Iron homeostasis as a pivotal factor not only influences innate immunity but also determines T-cell-mediated immune response (51). Proliferation and activation of T cells in the process of immune response (e.g., infection or tumor) will be delayed without iron. Activation of T cells is orchestrated by the uptake of iron through TfR1 (CD71) *via* IL-2-dependent pathway (52). Mutation of TfR1 leads to the impaired iron endocytosis and functional defects in T cells. This disorder finally leads to the occurrence of combined immunodeficiency disease (50). The number of mature T cells will decrease if ferritin H is knocked out in the bone marrow (53), which suggests that iron stored in ferritin is necessary for lymphocyte survival. Recent studies demonstrated that iron inhibits Th1 cells differentiation and expression of interferon-gamma (IFN-γ) (54). Besides, studies showed that iron inhibits Th1 lymphocyte activity (55). Some particles containing iron such as welding fume inhibit Th1 lymphocyte activity (55). However, interestingly, some adjuvants based on iron oxide nanoparticles promote Th1, Th17, and TCD8 immune responses, highlighting the role of iron as adjuvants in T-cell-mediated adaptive immunity (56). In summary, iron as an inherent factor in adaptive immunity inhibits Th1 cells differentiation and activity. However, on the other hand, iron as adjuvant promotes Th1 cells immune response. Similarly, Th2 cells differentiation and immune responses are suppressed by iron (27, 57). Notably, Ban et al. reported that iron had different impacts on Th2 cells immune response (suppressed or enhanced) due to the dose and size of iron particles (57). In Th17 cells, the role of iron remains controversial (27, 57–59). Some studies showed that iron attenuates Th17 activities and differentiation (27, 57), whereas other studies demonstrated that iron is indispensable for Th17 differentiation and pathogenicity (59). We speculate that the reason for those different phenotypes is due to the characteristic of different diseases (e.g., tumor and auto-immune disease) and the amount of iron used in each study. It is therefore meaningful to investigate the underlying mechanisms on those aspects. Few studies focused on the interactions between iron and Tfh cells; Yao et al. indicated that a new type of cell death named ferroptosis exists in Tfh cells; they found that inhibition of ferroptosis leads to increase in humoral immunity (60). Since ferroptosis is characterized by iron-dependent lipid peroxidation, future studies of exploring the underlying mechanisms and regulations of intracellular iron and lipid metabolism in Tfh cells will be interesting. Notably, as no study uncovers the links between iron and Tfh cells, it is promising that more work needs to be done in the future. In Treg cells, the expression of transferrin receptor 1 (CD71) is higher than that of CD4 ^+^ T cells (61). Upregulation of CD71 resulted in a higher intracellular iron transport, and this transportation in turn leads to the death of Treg (61). Accordingly, high level of iron may contribute to the death of Treg due to the imbalance of iron and ROS. As interactions among iron and Th1, Th2, Th17, Treg, and CTL are complicated, the specific role of iron in T-cell differentiation and immune response remains to be thoroughly investigated (**Table 3**). In conclusion, iron inhibits Th1, Th2, and Th17 cells differentiation and activity; however, iron as adjuvant promotes Th1 and Th2 cells immune response. Moreover, iron promotes CTL differentiation.

**Table 3:** Iron and T cells differentiation and activity.

T cell	Experimental model	Treatment	Major findings	Ref.
Th1	C57BL/6 infection mice	Low iron diet (≤9 mg), or high iron diet (5g/kg)	Iron inhibits Th1 cells differentiation by TIM-3	(54)
	Human respiratory tract infection	Human welding-fume exposure	Welding fume containing iron can inhibit Th1 lymphocyte activity.	(55)
	BALB/c mice (female, 4–8 weeks old)	Vaccinated subcutaneous	Iron as adjuvant promote Th1 cells immune responses	(56)
Th2	Tumor-bearing mice cryo-thermal therapy model	Cryo-thermal therapy	Iron inhibited the differentiation of Th2 cells.	(27)
	BALB/c mice (female, 7 weeks old)	Iron oxide in high or intermediate doses	Iron inhibits Th2 cell-mediated immune responses	(57)
	BALB/c mice (female, 7 weeks old)	Iron oxide nanoparticles	Iron as adjuvant promote Th2 cell-mediated immune responses	(57)
Th17	Tumor-bearing mice cryo-thermal therapy model	Cryo-thermal therapy	Iron inhibits Th17 cells differentiation.	(27)
	BALB/c mice (male, 5-6 weeks old)	Iron oxide nanoparticles (IONPs)	Iron inhibits activity of Th17 cells.	(62)
	DSS-induced colitis model, C57BL/6J mice	hemin	Hemin reduced the number of colon cancer Th17 cells; Hemin ameliorates dextran sodium sulfate-induced colitis	(59)
	Autoimmune encephalomyelitis, C57BL/6J mice at 6–8 weeks	N/A	Deficient of iron impair the function of Th 17 cells.	(58)
Treg	Systemic autoimmune disorders C57BL/6J condition KO mice	N/A	High level of iron may contribute to the death of Treg due to its imbalance of iron and ROS	(61)
CTL	Tumor-bearing mice cryo-thermal therapy model	Cryo-thermal therapy	Iron promoted the differentiation of CTL subsets	(27)

TIM, T cell immunoglobulin and mucin domain-containing protein 3; DSS, dextran sodium sulfate; CTL, cytolytic T lymphocyte; ROS, reactive oxygen species. N/A means Not Applicable.

## Iron and B Cells

Impairment and dysfunction of B cells due to the mutation of transferrin receptor 1 (TfR1) encoded by TFRC cause combined immunodeficiency (50). Iron, as an essential trace element, not only induced expression of cyclin E but also promoted proliferation of B cells (63). Lack of iron contributed to the downregulation of T-cell-mediated antibody response (63) and led to reduction in immune response. Recent studies indicated that serum iron levels are associated with antibody responses in human vaccination (63). Patients with iron deficiency showed a significantly decreased antibody response. In hematological cancer, antibodies targeting TfR1 (CD71) showed a potential therapy candidate in B-cell malignancy multiple myeloma (MM) (64). Interestingly, on the contrary, lactoferrin (LF), a pleiotropic iron-binding glycoprotein, as an iron chelator protein, not only stimulates B cells to produce IgA and IgG2b but also helps to protect host from pathogens invasion (65). Hence, these findings uncovered the complicated role of iron, as a double sword, in adaptive immunity and B cells.

## Iron and Immune Regulation in Diseases

Disturbance of iron homeostasis is common in infection, cancer, and autoimmune diseases. Pathophysiology or therapeutic regulation of iron has an impact on the outcome of those diseases (30).

## Iron and Immune Regulation in Infection

Pathogens grow fast under the condition of free iron in blood and thus promote pathogenicity to the host. Transportation of iron is regulated differently due to the nature of pathogen or the level of iron in cells. A fascinating phenomenon is that iron overload diseases such as thalassemia or primary hemochromatosis often lead to the host more susceptible to infection; in contrast, modest iron deficiency can prevent malaria (66). A decrease in iron levels in the plasma could be observed due to the secretion of hepcidin when infection or inflammatory injury occurs. However, a low level of iron in blood limits the utilization of iron in pathogens; on the other hand, it also limits the development of RBCs in the bone marrow and thus led to inflammatory anemia (67). Recently, Rochette et al. found oxidative stress and inflammatory responses due to dysregulation of iron homeostasis in coronavirus disease 2019 (COVID-19) patients (68). Disturbance of iron homeostasis, elevated iron levels, in particular, may contribute to the progression of viral infection. They claimed that serum ferritin level is correlated with COVID-19, and it may be helpful in predicting the outcomes (68). In conclusion, the regulation of systemic iron levels acts as an immune-responsive role in host infection.

## Iron and Immune Regulation in Cancer

One of the metabolic markers that malignant tumors have is the disorder of iron metabolism due to its high metabolic activity (69). ROS induced by iron followed by the damage of DNA, lipid, and protein in normal cells leads to tumorigenesis (70, 71). Moreover, emerging evidence demonstrates that iron is involved in tumor development, metastasis, and tumor microenvironment (TME) modification (72).

Currently, as a novel anti-cancer strategy, using immunotherapy-induced autoimmune system to find out and destroy tumor has become promising. Hence, understanding the interaction among iron, immune system, and cancer seems to be important. In lung cancer, tumor-associate macrophage (TAM) containing iron has been demonstrated to increase ROS level and secrets proinflammatory cytokines (e.g., TNFα, IL-6) to kill tumor cells (73). Indeed, the iron supplement, ferumoxytol, a compound for the treatment of iron deficiency anemia in chronic kidney disease approved by FDA, suppresses the growth of early-stage breast cancer and metastasis of lung cancer by M1 macrophages (74). Moreover, ferumoxytol protects the liver from metastatic lesions invading and increases macrophage function in cancer immunotherapy (74). However, on the contrary, TAM itself as an “iron donor” that releases iron into cancer microenvironment promotes cancer progression (75, 76), and TAM provides iron to tumor by Lipocalin-2 (LCN-2) (77, 78). Based on those findings, iron chelators were therefore used in anti-tumor therapy. Deferoxamine and deferasirox, as two of the iron chelators approved by the Food and Drug Administration (FDA), have been proved to be effective in several types of cancer such as leukemia, neuroblastomas, colorectal, pancreatic, and breast cancer (79–83). Accordingly, the specific role of iron in TAM is controversial; more studies are needed to investigate the underlying mechanisms and the relationship between iron and tumor-associated macrophages in cancer and its “iron donor” role in the progression of cancer. In addition, crosstalk between iron and TAM in tumor also requires to be further investigated.

## Iron and Immune Regulation in Aging Diseases

Cell senescence is characterized by irreversible proliferation arrest, secretion of proinflammatory cytokine, and escape of programmed cell death. Aging changes the acquisition and storage of iron and thus leads to the change in intracellular iron level (84). Iron is accumulated in replicative aging fibroblasts *in vitro* (85); moreover, intracellular ferritin is enriched in aging tissues (86). Intracellular iron homeostasis acts as a “messenger” to communicate between lysosomes and mitochondria under inflammatory conditions (87–89). Indeed, changes in mitochondrial membrane potential and the use of iron chelators reduce synthesis of interleukin (IL-1β) in macrophages (28, 90). Macrophages iron overload induces p16^INK4a^-dependent aging process in resident fibroblasts, and this eventually leads to the delay of wound healing (18). Thus, targeting iron in macrophages provides us a novel perspective in the treatment of immune and aging disease.

## Conclusion

Collectively, as a critical element for living cells, iron is capable of regulating both innate and adaptive immunity. Specifically, iron regulates macrophage polarization; the majority of the research indicated that iron promotes macrophage M1 polarization, whereas some demonstrated that iron promotes M2 polarization. In neutrophils, iron regulates neutrophils recruitment and inflammation. Iron-deficient environment promotes the formation of]NETs. Transferrin secreted by human neutrophils promotes tumor metastasis. In T cells, iron inhibits Th1, Th2, and Th17 cells differentiation and activity; however, iron as adjuvant promotes Th1 and Th2 cells immune response. Moreover, iron promotes CTL differentiation. Based on the previous findings in basic research, some strategies targeting iron have been shown to be a promising alternative in several diseases such as infectious, COVID-19, aging diseases, and cancer. However, the specific mechanisms of iron in immune regulation especially in TAM remains partly unclear. The outcomes of these therapeutic strategy targeting iron requires to be further investigated in the future.

## Author Contributions

SN and YY contributed equally in this work. SN and YY conceived the manuscript, XL and YK designed the project and revised the manuscript. All authors contributed to the article and approved the submitted version.

## Funding

This work was supported by the National Natural Science Foundation of China (Grant No. 82072422, 82003777).

## Conflict of Interest

The authors declare that the research was conducted in the absence of any commercial or financial relationships that could be construed as a potential conflict of interest.

## Publisher’s Note

All claims expressed in this article are solely those of the authors and do not necessarily represent those of their affiliated organizations, or those of the publisher, the editors and the reviewers. Any product that may be evaluated in this article, or claim that may be made by its manufacturer, is not guaranteed or endorsed by the publisher.

## Glossary

**Table:**

NK cells	natural killer cells
Th cells	T helper cells
CTL	cytotoxic T lymphocyte
ROS	reactive oxygen species
PRR	pattern recognition receptors
PAMP	pathogen-associated molecular patterns
DAMP	danger-associated molecular patterns
TNF	tumor necrosis factor
CD	cluster of differentiation
FAC	ferric ammonium citrate
BMDM	bone-marrow-derived macrophages
RBC	red blood cells
PEG	polyethylene glycol
PEG-Fns	PEG-coated ferrihydrite nanoparticles
DFE	deferiprone
MPO	myeloperoxidase
FPN	ferroportin
CXCL1	chemokine (C-X-C motif) ligand 1
DFO	deferoxamine
NET	neutrophil extracellular traps
TRAIL	tumor necrosis factor (TNF)-related apoptosis-inducing ligand
FasL	Fas ligand
IFN	interferon
FTH	ferritin H
MHC	major histological complex
TfR1	transferrin receptor 1
TCD8	T-cell CD8^+^
Tfh	T follicle helper cells
Treg	T regulatory cells
TIM3	T-cell immunoglobulin and mucin domain-containing protein 3
DSS	dextran sodium sulfate
MM	multiple myeloma
TME	tumor microenvironment modification
TAM	tumor-associated macrophage
